# Green Vehicle-Routing Problem of Fresh Agricultural Products Considering Carbon Emission

**DOI:** 10.3390/ijerph19148675

**Published:** 2022-07-16

**Authors:** Qi Yao, Shenjun Zhu, Yanhui Li

**Affiliations:** 1 Management School, Wuhan College, Wuhan 430212, China; yaoqi@mails.ccnu.edu.cn; 2 School of Information Management, Central China Normal University, Wuhan 430079, China; sjzhu@mails.ccnu.edu.cn

**Keywords:** vehicle-routing problem, fresh agricultural products, carbon emission, green distribution, improved ant-colony optimization

## Abstract

The need to reduce carbon emission to cope with climate change has gradually become a global consensus, which also poses a great challenge to cold-chain logistics companies. It forces them to implement green distribution strategies. To help the distribution companies reduce carbon emission, this paper studies two aspects—carbon tax value and investing in the freshness-keeping cost—and proposes corresponding solutions. A new green vehicle-routing model for fresh agricultural products with the goal of minimizing the total cost is proposed. To solve the model proposed, an improved ant-colony optimization (IACO) is designed specifically. On one hand, the experimental results show that the increase in carbon tax will restrict the carbon emission behaviors of the distribution companies, but it will also reduce their economic benefits to a certain extent, at the same time. On the other hand, investing in the freshness-keeping cost can help actively achieve the carbon emission reduction target, reduce the loss of fresh agricultural products in the distribution process, improve the company’s economic benefits and satisfy customers. The comparison results of different algorithms prove that the IACO proposed in this paper is more effective in solving the model, which can help increase the economic benefits of the companies and reduce carbon emission. This study provides a new solution for cold-chain logistics distribution companies to reduce carbon emission in the distribution process, and also provides a reference for government departments to formulate carbon tax policies.

## 1. Introduction

Since human society entered the era of industrialization, greenhouse gas emissions dominated by CO_2_ have increased rapidly, and the concentration of CO_2_ and other greenhouse gases has been rising. This phenomenon is likely to be the main cause of climate change [1,2]. The governments and management departments of various countries have taken some measures as a result, such as introducing carbon emission trading mechanisms, carbon tax and other policies. Many companies have also made green and sustainable-development strategies to reduce carbon emission in cold-chain logistics. Reducing carbon emission and implementing green distribution strategies is vital to cold-chain distribution companies. As a new force in the field of transportation, cold-chain distribution companies play an important role in ensuring food hygiene and safety for people’s livelihoods. At the same time, they are also facing severe pressure regarding carbon emission reduction and environmental protection. On the other hand, with the development of society and the improvement of people’s living standards, people’s demand for fresh agricultural products is constantly “upgrading”.

Devapriya et al. studied the problem of unifying the production and distribution scheduling of fresh agricultural products, and designed an accurate algorithm to solve the problem [3]. Wang et al. proposed a vehicle-routing problem in cold-chain logistics with time window constraints, which takes controlling and limiting carbon emission and fuel consumption as the main objective [4]. Chen et al. studied the distribution of a variety of fresh agricultural products, formed a multi-chamber vehicle-routing problem, and established a cold-chain logistics distribution problem model with the objective of minimizing the total cost [5]. The minimum freshness requirement of customers has also been considered, and a two-level programming mathematical model was solved to optimize the cold-chain logistics distribution problem [6]. In view of the problem of high corruption and damage rates of fresh agricultural products occurring during the distribution process, related research has given some solutions. For example, the cold-chain food distribution plan was adopted to reflect the law of the decline of food quality over time [7]. An IoT (Internet of Things)-based route-planning system (IRPS) can reduce food loss during transportation and the time required to deliver spoiled food, which helps to improve customer satisfaction [8]. An intelligent measuring device was used to calculate the residual value of fresh agricultural products in the transportation process in real time and match the estimated remaining shelf life with the expected remaining transportation time, so as to improve the net present value (NPV) [9]. With the goal of controlling fuel consumption and minimizing the total transportation cost, Wei et al. provided a new solution for the inventory routing problem of China’s cold chain logistics industry [10].

The distribution process is one of the cores of the cold-chain logistics system, and has also been the focus of related research in recent years. With the increasingly serious global environmental problems, the proposal of energy saving and carbon emission reduction targets, more and more scholars who study cold-chain logistics have included carbon emission in the total cost and put forward a series of new ideas for improving environmental problems. In order to help control carbon emission, the cost of carbon emission was also included in the objective function, so a green vehicle-routing model of cold-chain logistics was constructed [11]. Qin et al. comprehensively considered the objective of carbon emission and customers’ satisfaction. They constructed a new model and analyzed the impact of carbon tax on carbon emission and average customer satisfaction [12]. Babagolzadeh et al. established a two-stage stochastic programming model on the premise of carbon tax regulation and uncertain demand to determine the optimal replenishment strategy and transportation plan [13]. They also concluded that carbon emission can be controlled to minimize the total cost. Wang et al. proposed a vehicle-route problem for cold-chain logistics with time windows based on carbon tax. Through experimental data, they analyzed the difference in carbon emission, the change in distribution path, and the effect of carbon tax on total cost under different carbon tax conditions [14]. Leng et al. proposed a two-layer optimization model for cold-chain logistics to minimize the total distribution cost and waiting times of the customers and vehicles. Their first target was to minimize the total distribution cost, which included the fixed cost of opening the warehouse, the vehicle leasing cost, the fuel consumption cost, the carbon emission cost and the cost of damage of goods. Additionally, the second objective included the waiting time [15]. Liu et al. combined multiple cold-chain logistics companies to establish a joint distribution model for green vehicle-routing problems. By considering carbon tax policies to deliver cold-chain products, it proved that joint distribution is an effective way to reduce total distribution costs and control carbon emission [16].

VRP is a classic combinatorial optimization problem. Since it was first proposed by Dantzig and Ramser [17], it has received extensive attention from researchers. Braekers et al. gives a good overview and classification of VRP [18]. Since VRP is an NP-hard problem, researchers usually use heuristic and meta-heuristic methods to solve them. These include ant-colony optimization [19,20,21], the genetic algorithm [22,23], the tabu search algorithm [24,25], the simulated annealing algorithm [26,27], the iterative local search algorithm (ILS) [28], the multi-group PSO method [29] and the large-neighborhood search [30,31]. In this paper, we use an improved ant-colony optimization to solve the model we proposed. Ant-colony optimization (ACO) has the characteristics of a positive feedback mechanism and strong robustness. ACO is widely used to solve VRP and other combined optimization problems such as traveling salesman problems [32], scheduling problems [33], mobile robot path-planning problems [34] and cloud computing problems [35].

Relevant studies have described, in detail, the cost of each part of the cold-chain logistics distribution company in the distribution process and their relationships with each other. However, these have some limitations. On one hand, many scholars take the economic benefits of the distribution company as the only goal. They do not take the pollution of carbon emission to the environment into consideration, nor do they consider restricting carbon emission through carbon tax policies. On the other hand, few combine the vehicle-routing problems with controlling cargo damage in the distribution process. In addition to the significant increase in the demand for fresh agricultural products, the requirements for their quality, especially for freshness, are becoming more and more stringent. All of these points have brought new challenges to cold-chain logistics companies, and also need more attention. Many may not realize that the behavior for reducing carbon emission and controlling cargo damage are often inseparable. Most companies only take the time window constraints required by customers into consideration, while not improving customers’ satisfaction from the perspective of reducing cargo damage and improving the freshness of the fresh agricultural products they need. Based on the above two limitations, the content of this paper is presented from two aspects. First, the effect of different carbon tax amounts on the change in carbon emission is analyzed. Then, the vehicle-routing problem of cold-chain logistics is combined with the control of cargo damage in the distribution process, and the minimum freshness requirement constraint of customers is also considered. A new VRP model of cold-chain logistics is built, and the methods companies can use to control carbon emission are also explored. Besides, the new VRP model in this paper is very difficult to solve, which is a typical NP-hard problem. Therefore, this paper adopts an improved ant-colony algorithm, which is very efficient for solving the VRP model.

## 2. Problem Description and Model Formula

### 2.1. Problem Description

The fresh agricultural products mainly include fresh primary products such as vegetables, fruits, flowers, meat, eggs, milk and aquatic products, which are perishable and vulnerable. The cold-chain logistics system is often used in the transportation and distribution of fresh agricultural products. The distribution system studied in this paper is composed of a single distribution center and multiple customers. All the vehicles start from a distribution center and return to the same distribution center after completing the distribution tasks. The distribution center delivers only one type of fresh agricultural product to all customers. Consumers often have certain freshness requirement constraints for the fresh agricultural products they need. Therefore, cold-chain companies need to consider keeping the agricultural products fresh in the distribution process to meet customers’ requirements for freshness. The green distribution strategy is a realistic choice for the companies. The green distribution strategy consists of choosing a reasonable distribution route, effectively using vehicles, reducing distribution costs and resource consumption, and reducing carbon emission. The objective of the system is to minimize the total distribution cost. Referring to the relevant literature, the following assumptions are put forward:

(1) The distribution center has a sufficient number of vehicles of the same type. The maximum load of all the vehicles is determined.
(2) The demand of each customer is less than the maximum load of the vehicle, and it is only satisfied by one vehicle.
(3) The coordinates, demand, time window and service time of each customer are known. The distance between any two customers is calculated by the straight-line distance. The customers have certain freshness constraints for the fresh agricultural products they need.
(4) The vehicles are allowed to arrive earlier or later than the time window required by the customers, but the companies need to pay the penalty cost.
(5) The vehicles only provide distribution services for fresh agricultural products, including driving and unloading.
(6) There is no significant difference in the driving skills and operating proficiency of all the drivers, and the influences of subjective factors on vehicle speed and fuel consumption are not considered.

### 2.2. Symbol Definitions

(1) Decision variables

In order to facilitate modeling below, we set the number of the distribution center to 0. Each customer is represented by characters *i* and *j* (*i*, *j* = 1, 2, …, *N*), and the route between customers *i* and *j* is denoted as (*i*, *j*). The values and descriptions of the decision variables are as follows:

*x_ijk_*: Has a value of 1 when the vehicle *k* goes from customer *i* to *j*. Otherwise it has a value of 0.

*x*_0*jk*_: Has a value of 1 when the company assigns vehicle *k* to complete the distribution task. Otherwise it has a value of 0.

*y_ik_*: Has a value of 1 when the demand of customer *i* is satisfied by the vehicle *k*. Otherwise it has a value of 0.

(2) Parameters

The descriptions of the parameters are as follows:

*N*: The number of customers that need the service from the distribution center.

*K*: The number of the vehicles available at the distribution center.

*f_k_*: The fixed cost of using each vehicle.

*Q*: The maximum load capacity of the vehicles.

*Q_ij_*: The cargo capacity of the vehicle from customer *i* to *j*.

*Q_in_*: The weight of the vehicle when it leaves customer *i*.

*q_i_*: The quantity of fresh agricultural products required at customer *i*.

*P*: Unit price of fresh agricultural products.

*c_fuel_*: Unit price of the fuels.

*υ*: Carbon tax that the companies need to pay for carbon emission.

*ω*: Carbon emission factor.

*a*: The consumption coefficient of the refrigerant during the driving process of the vehicle.

*b*: The consumption coefficient of the refrigerant during the unloading process of the vehicle.

*η*_1_: The freshness attenuation coefficient of fresh agricultural products without freshness investment during the driving process.

*η*_2_: The freshness attenuation coefficient of fresh agricultural products without freshness investment during the unloading process.

$t_{i}^{k}$: The time point at which the vehicle *k* arrives at customer *i*.

$t_{0}^{k}$: The time point at which the vehicle *k* departs from the distribution center.

*d_ij_*: The distance between customers *i* and *j*.

*C_f_*: The freshness-keeping cost of the fresh agricultural products invested by the company per unit time and unit weight during the distribution process.

*T_i_*: The service time of the vehicle to customer *i*.

*ε*_1_: The default cost factor for the vehicle if arriving earlier than the required time period of the customer.

*ε*_2_: The default cost factor for the vehicle if arriving later than the required time period of the customer.

### 2.3. Relevant Parameters

#### 2.3.1. Changes in Freshness

The main factors leading to the damage of goods during the distribution process include the distribution process, the nature of fresh agricultural products and the distribution time span. The decay law of fresh agricultural products in the environment of refrigerated vehicles with time changes can be expressed as:

(1) $$\theta_{t}=\theta_{0}e^{-\eta t}$$

where *θ_t_* is the freshness of the fresh agricultural products at time point *t*; *θ*_0_ is the freshness of the fresh agricultural products at the initial time; and *η* is the freshness attenuation coefficient of the fresh agricultural products.

Without investing in the freshness-keeping cost, the freshness over time is:

(2) $$F_{i}=e^{-\eta (t_{i}^{k}-t_{0}^{k})}$$

where *F_i_* is the freshness of the fresh agricultural products when the vehicle *k* arrives at customer *i*.

According to the research of Chen et al. [36], the input of the freshness-keeping cost (*C_f_*) can reduce the freshness attenuation coefficient of fresh agricultural products from *β_f_* to *η*/(1 + *β_f_C_f_*). The larger the value of *β_f_*, the easier it is for the fresh agricultural products to be preserved during the distribution process. Therefore, under the condition of freshness-keeping cost invested, the freshness over time is:

(3) $$F_{i}=e^{-\frac{\eta }{1+\beta_{f}C_{f}}(t_{i}^{k}-t_{0}^{k})}$$

#### 2.3.2. Selection of Vehicle

Since the load of the vehicle is determined, and the customers have certain freshness requirement constraints of their fresh agricultural products, the vehicle *k* needs to consider both factors when choosing the next customer *j* after completing the distribution task at customer *i*:

(4) $$\left\{ \begin{array}{ll} \sum_{k=1}^{i}q_{k}+q_{j}\leq Q, & \forall j\;\\ F_{j}>F_{d}, & \forall j \end{array}\right.$$

In Formula (4), the first expression is the vehicle’s load constraint, and the second expression is the customer’s freshness requirement constraint. If the vehicle meets the two constraints at the same time, it will go straight to customer *j* and complete the delivery task. Otherwise, it will return to the distribution center. Additionally, the company needs to reassign another vehicle to continue the delivery task.

### 2.4. Component of Total Cost

#### 2.4.1. Fixed Cost (*C*_1_)

When the distribution center assigns a vehicle to deliver fresh agricultural products, it has to pay a certain fixed cost. It is mainly composed of the driver’s salary, the cleaning and maintenance cost of the vehicle, and the depreciation cost. According to assumptions (1) and (6), this part of the cost is only related to the number of vehicles to be used, which can be expressed by Formula (5):

(5) $$C_{1}=\sum_{k=1}^{K}\sum_{i=1}^{N}x_{0ik}f_{k}$$

#### 2.4.2. Fuel Consumption Cost and Carbon Emission Cost (*C*_2_)

The fuel consumption is related to the transportation distance and load capacity of the vehicle, and the fuel consumption cost is calculated using the load estimation method [37]. Through the regression analysis result of a large number of statistical data, this method draws the conclusion that there is a linear related relationship between the fuel consumption *ρ* per unit distance and the cargo weight *X* of the vehicle. The weight of the vehicle is divided into the self-weight *Q*_0_ and the cargo weight *X*. The expression of fuel consumption per unit distance *ρ*(*X*) of the vehicle under the condition of loading *X* is shown in Formula (6):

(6) $$\rho (X)=p(Q_{0}+X)+q$$

The maximum load capacity of the vehicle is set as *Q*, the fuel consumption per unit distance of driving is *ρ*_0_ when the vehicle is empty, and it is ρ_*_ when the vehicle is fulyl loaded. According to Formula (6), the expressions of *ρ*_0_ and ρ_*_ can be obtained as follows:

(7) $$\rho_{0}=pQ_{0}+q$$

(8) $$\rho_{*}=p(Q_{0}+Q)+q$$

where *p* and *q* are two known parameters, and the expression for *p* obtained from Formulas (7) and (8) is:

(9) $$p=\frac{\rho_{*}-\rho_{0}}{Q}$$

Therefore, the fuel consumption of per-unit distance *ρ*(*X*) when the cargo load is *X* can be expressed as:

(10) $$\rho (X)=\rho_{0}+\frac{\rho_{*}-\rho_{0}}{Q}X$$

The fuel consumption *fuel_ij_* of the vehicle from customer *i* to *j* is:

(11) $$fuel_{ij}=\rho (Q_{ij})d_{ij}$$

where *ρ*(*Q_ij_*) is the fuel consumption rate when the vehicle travels directly from customer *i* to *j* with load *Q_ij_*, and *d_ij_* is the distance between customer *i* and *j*.

When all vehicles complete the delivery tasks to the customers and return to the distribution center, the total fuel consumption is:

(12) $$fuel=\sum_{k=1}^{K}\sum_{i=1}^{N}\sum_{j=1}^{N}x_{ijk}fuel_{ij}$$

The total fuel consumption cost *C*_21_ generated in the distribution process is:

(13) $$C_{21}=c_{fuel}fuel$$

Carbon emission mainly refers to the fuel consumption during the distribution process. The research shows that there is a certain linear relationship between carbon emission and fuel consumption [38]. That is, carbon emission can be obtained by multiplying the fuel consumption and carbon emission factors. Therefore, the carbon emission cost *C*_22_ paid by the companies in the entire distribution process can be obtained as follows:

(14) $$C_{22}=\upsilon \omega fuel$$

The fuel consumption cost and carbon emission cost generated during the distribution process is:

(15) $$C_{2}=C_{21}+C_{22}=(c_{fuel}+\upsilon \omega )fuel$$

#### 2.4.3. Refrigeration Cost and Freshness-Keeping Cost (*C*_3_)

The refrigeration cost is the cost of the refrigerant (ice cubes, liquid nitrogen, etc.) required to maintain the low temperature conditions inside the vehicle during the distribution process. The amount of refrigerant used is approximately positively correlated with the driving time of the vehicle. Its expression is:

(16) $$C_{31}=\sum_{k=1}^{K}\sum_{i=1}^{N}\sum_{j=1}^{N}\left(at_{ij}^{k}x_{ijk}+bT_{i}y_{ik}\right)$$

where $t_{ij}^{k}$ is the travel time of vehicle *k* between customer *i* and *j*, and *T_i_* is the service time of customer *i*.

The freshness-keeping cost is the cost invested in the distribution process for the purpose of slowing down the spoilage and deterioration rate of fresh agricultural products. It mainly includes the cost of relevant freshness-keeping packaging and chemical preservatives. Its expression is:

(17) $$C_{32}=\sum_{k=1}^{K}\sum_{i=1}^{N}\sum_{j=1}^{N}\left[C_{f}\frac{Q_{ij}}{100}(t_{ij}^{k}x_{ijk}+T_{i}y_{ik})\right]$$

where *C_f_* is the investment in the freshness-keeping cost by the distribution companies for the fresh agricultural products per unit time, and per hundred unit weight during the distribution process.

Therefore, the refrigeration cost and freshness-keeping cost *C*_3_ invested by the companies for fresh agricultural products are:

(18) $$C_{3}=C_{31}+C_{32}=\sum_{k=1}^{K}\sum_{i=1}^{N}\sum_{j=1}^{N}\left[(a+C_{f}\frac{Q_{ij}}{100})t_{ij}^{k}x_{ijk}+(b+C_{f}\frac{Q_{ij}}{100})T_{i}y_{ik}\right]$$

#### 2.4.4. Cargo Damage Cost (*C*_4_)

According to the analysis in Section 2.3.1, the expression of the cargo damage cost *C*_4_ caused during the distribution process is:

(19) $$C_{4}=\sum_{k=1}^{K}\sum_{i=0}^{N}y_{ik}P\left[q_{i}\left(1-e^{-\frac{\eta_{1}}{1+\beta_{f}C_{f}}(t_{i}^{k}-t_{0}^{k})}\right)+Q_{in}(1-e^{-\frac{\eta_{2}}{1+\beta_{f}C_{f}}T_{i}})\right]$$

#### 2.4.5. Penalty Cost (*C*_5_)

In reality, the customers will require the company to deliver the required fresh agricultural products within the specified time window (*L_i_*, *R_i_*) due to its own operational needs. According to assumption (5), if the vehicle arrives at the customer earlier or later than the specified time window indicates, the companies must pay penalty costs for violations of the time window. The time window penalty cost in the entire delivery process is:

(20) $$C_{5}=\varepsilon_{1}\sum_{i=1}^{N}\max\{L_{i}-t_{i},0\}+\varepsilon_{2}\sum_{i=1}^{N}\max\{t_{i}-R_{i},0\}$$

where *t_i_* is the actual time at which the vehicle arrives at customer *i*.

### 2.5. Model Formula

Based on the analysis in Section 2.4, the VRP model for the company under the condition of investing in the freshness-keeping cost is given by the following.

(1) Objective function
  (21) $$\begin{array}{l} \min Z_{1}=C_{1}+C_{2}+C_{3}+C_{4}+C_{5}=\\ \sum_{k=1}^{K}\sum_{i=1}^{N}x_{0ik}f_{k}+(c_{fuel}+\upsilon \omega )fuel+\\ \sum_{k=1}^{K}\sum_{i=1}^{N}\sum_{j=1}^{N}\left[(a+C_{f}\frac{Q_{ij}}{100})t_{ij}^{k}x_{ijk}+(b+C_{f}\frac{Q_{ij}}{100})T_{i}y_{ik}\right]+\\ \sum_{k=1}^{K}\sum_{i=0}^{N}y_{ik}P\left[q_{i}\left(1-e^{-\frac{\eta_{1}}{1+\beta_{f}C_{f}}(t_{i}^{k}-t_{0}^{k})}\right)+Q_{in}(1-e^{-\frac{\eta_{2}}{1+\beta_{f}C_{f}}T_{i}})\right]+\\ \varepsilon_{1}\sum_{i=1}^{N}\max\{L_{i}-t_{i},0\}+\varepsilon_{2}\sum_{i=1}^{N}\max\{t_{i}-R_{i},0\} \end{array}$$
(2) Constraints
  (22) $$F_{i}>F_{d},\forall i$$
  (23) $$\sum_{i=1}^{N}q_{i}y_{ik}\leq Q_{k},\forall k$$
  (24) $$\sum_{k=1}^{K}y_{ik}=1,\forall i$$
  (25) $$\sum_{k=1}^{K}\sum_{j=0}^{N}x_{0jk}=\sum_{k=1}^{K}\sum_{j=0}^{N}x_{j0k}$$
  (26) $$\sum_{j=0}^{N}x_{ijk}=y_{jk},\forall i,k$$
  (27) $$\sum_{i=0}^{N}x_{ijk}=y_{ik},\forall j,k$$
  (28) $$\sum_{i,j\in S\times S}^{N}x_{ijk}\leq |S|-1,\;S\subseteq \left\{1,2,\ldots ,N\right\}$$
  (29) $$t_{j}=t_{i}+T_{i}+t_{ij},\forall i,j$$
  (30) $$x_{ijk},y_{ik}=0\;or1,\forall k,i,j$$

Formula (22) is the freshness requirement constraint of the customers. Formula (23) is the demand constraint of every customer. Formula (24) indicates that each customer’s demand can only be completed by one vehicle. Formula (25) indicates that the vehicle starts from the distribution center to complete the distribution. After finishing all the tasks, it will finally return to the distribution center. Formulas (26) and (27) indicate that the vehicle is only allowed to depart from and arrive at any customer only once. Formula (28) is to eliminate the secondary loop in the distribution process. Formula (29) ensures that the distribution process is continuous. Formula (30) is to constrain the decision variables’ value.

## 3. Improved Ant-Colony Optimization (IACO) Design

The VRP model established in Section 2.5 is a mixed-integer programming (MIP) model, which is a class of NP-hard problem and has great difficulty in solving. In this paper, an improved ant-colony optimization (IACO) is designed to solve the VRP model. The traditional ant-colony optimization algorithm has been improved, and the heuristic factor, ant state-transition strategy and pheromone-update mechanism have been redesigned to make it more suitable for solving the VRP model. The method proposed in this paper makes better use of the positive feedback and parallel search characteristics of the ant-colony optimization to gradually find the global optimal path.

### 3.1. Heuristic Factor Design

The design of the heuristic factor *η_ij_* is vital for the ACO to find the optimal solution. A suitable heuristic factor can better guide the ants to choose the next customer. In the traditional ACO, the distance between two customers is the only consideration. However, the objective of the model is to minimize the total distribution cost. In the process of vehicle distribution, in addition to the distance, another important factor that determines the total cost is the load of the vehicle; this is because there are parameters related to load in the expressions of carbon emission and fuel consumption cost, cargo damage cost, refrigeration, and freshness-keeping cost. Therefore, a heuristic factor that comprehensively considers distance and vehicle load is designed in this paper:

(31) $$\eta_{ij}=\frac{1}{d_{ij}T_{j}}$$

### 3.2. State-Transition Strategy

The moving probability of ant *k* is:

(32) $$p_{ij}^{k}=\left\{ \begin{array}{ll} \frac{\tau_{ij}^{\alpha }\eta_{ij}^{\beta }}{\sum_{s\in J_{k}(i)}\tau_{is}^{\alpha }\eta_{is}^{\beta }}, & j\in allowed_{k}\\ 0, & \mathrm{otherwise} \end{array}\right.$$

where *J_k_*(*i*) is the set of customers that can be selected by the first ant after passing through customer *i*. *α* and *β* are two adjustable parameters. The values of *α* and *β* indicate the degree to which the pheromone and heuristic factor accumulated on the path can be selected by the ants in the process of selecting the next customer.

The following path selection rules are used to select the next customer to transfer. It can help prevent the ant-colony optimization from prematurely converging, falling into a local optimum and resulting in stagnation.

(33) $$j=\left\{ \begin{array}{ll} \underset{s\in J_{k}(i)}{\arg\max}\left\{\tau_{is}^{\alpha }\eta_{is}^{\beta }\right\}, & q_{random}\leq q_{0}\\ J, & q_{random}>q_{0} \end{array}\right.$$

Here, *q_random_* is a random variable; *q*_0_ (0 ≤ *q*_0_ ≤ 1) is uniformly distributed in the interval [0, 1]; and the parameter set *J* is the random customers generated according to the probability distribution given by Formula (32).

### 3.3. Pheromone-Updating Mechanism

In order to enable the updating of the pheromone to better guide the ants who are searching later, when all the ants in each generation complete their path searches, only the pheromone on the global optimal path is increased. The formula for increasing the pheromone is:

(34) $$\Delta \tau_{ij}^{best}(t)=\sum_{k=1}^{M}\Delta \tau_{ij}^{m}(t)$$

(35) $$\Delta \tau_{ij}^{m}(t)=\left\{ \begin{array}{ll} \frac{Q_{m}}{L_{best}(t)}, & \mathrm{if}\;\mathrm{ant}\;m\;\mathrm{passes}\;\mathrm{through}\;(i,\;j)\\ 0, & \mathrm{otherwise}\; \end{array}\right.$$

where $\Delta \tau_{ij}^{best}\left(t\right)$ is the total amount of pheromone added to the edge which is on the global optimal path after the iteration; *t* is the pheromone released by ant *m* on the edge on the global optimal path in this iteration; $\Delta \tau_{ij}^{m}\left(t\right)$ is the total amount of pheromone released by each ant; *M* is the total number of ants; and *L_best_* (*t*) is the length of the global optimal path after *t* iterations.

After *t* iterations, the pheromone-update formula for edges on the global optimal path is:

(36) $$\tau_{ij}(t)=(1-\rho )\tau_{ij}(t-1)+\Delta \tau_{ij}^{best}(t)$$

The pheromone-update formula for edges that are not on the globally optimal path is:

(37) $$\tau_{ij}(t)=(1-\rho )\tau_{ij}(t-1)$$

In Formulas (36) and (37), the parameter *ρ* is the pheromone volatilization coefficient. The value of *ρ* will also affect the convergence speed of the optimization process. A larger value of *ρ* at the beginning will lead the ants to search for the global optimal solution, and it should be reduced as the optimization progress goes on to help search for local optimal solutions. Therefore, the value of *ρ* changes with the increase in iterations as follows:

(38) $$\rho =\left\{ \begin{array}{l} 0.8,\;NC\in [0,NC_{\max}/5]\\ 0.5,\;NC\in (NC_{\max}/5,NC_{\max}/3]\\ 0.3,\;NC\in (NC_{\max}/3,NC_{\max}/2]\\ 0.1,\;NC\in (NC_{\max}/2,NC_{\max}] \end{array}\right.$$

where *NC* is the current number of iterations and *NC*_max_ is the maximum number of the ant-colony optimization’s iterations.

### 3.4. The Pseudo-Code Framework Diagram of IACO

The algorithm flow of IACO is shown in Algorithm 1.
**Algorithm 1. IACO for the Model**
(i) **Input:** each customer’s coordinates, demand, time window, service time, etc.
(ii) **Output:** best routine (*BR*), each part of the minimal total cost (*PMIN*) and the minimal total cost (*MIN*).
(1) Set *N*, *T*[*k*], *F*(*d*), *NC*_max_, *NC*, etc.
(2) Set *m* as the number of the ants.
(3) *NC* = 1
(4) **while**
*NC* ≠ *NC*_max_
(5)   Initialize the ant path and the pheromone of each path. Insert 0 into *T*[*k*]. All ants return to the distribution center.
(6)   **for**
*k* = 1: *m*
(7)    **while**
*N* ≠ *ϕ*
(8)     Select next customer *i* according to decision-making conditions. Calculate the cargo weight *Q*(*k*) and the freshness *F*(*k*) if ant *k* arrives at customer *i*.
(9)     **if**
*Q*(*k*) ≤ *Q and F*(*k*) ≥ *F*(*d*)
(10)      Insert customer *i* into *T*[*k*], delete *i* from *N*.
(11)     **else**
(12)      Ant *k* returns to the distribution center. Insert 0 into *T*[*k*]. *Q*(*k*) = 0, *F*(*k*) = 100%.
(13)     **end if**
(14)    **end while**
(15)   **end for**
(16)   Calculate the total cost according to *T*[*k*]. Select the best routine (*BR_n_*), each part of the minimal total cost (*PMIN_n_*) and the minimal total cost for this iteration (*MIN_n_*).
(17)   Update the pheromone of each path according to the best routine.
(18)   **if**
*MIN_n_* < *MIN*
(19)    *MIN* = *MIN_n_*. *BR* = *BR_n_*. *PMIN* = *PMIN_n_*.
(20)   **end if**
(21)   *NC* = *NC* + 1
(22) **end while**
(23) **return**
*BR*, *PMIN*, *MIN*

## 4. Experiments and Results

### 4.1. Example and Parameter Setting

The example R108 of VRPTW-type data in the Solomon Database [39] is selected as the simulation experimental data. It includes one distribution center and 50 customers. According to the actual situation, the unit of the customer’s demand and vehicle’s load in the calculation example is set to 10 kg, and the vehicle’s speed is 40 km/h.

The values of the related parameters in the model and IACO are shown in Table 1.

### 4.2. Effect of Carbon Tax on Carbon Emission

Referring to carbon taxes in developed and developing countries, the value of carbon tax is set from C0 from 0 to 10. Carbon emission, carbon emission cost (*C*_22_), and the total cost (TC) under different carbon tax amounts will be obtained. The experimental results are shown in Table 2.

**Figure 1:**
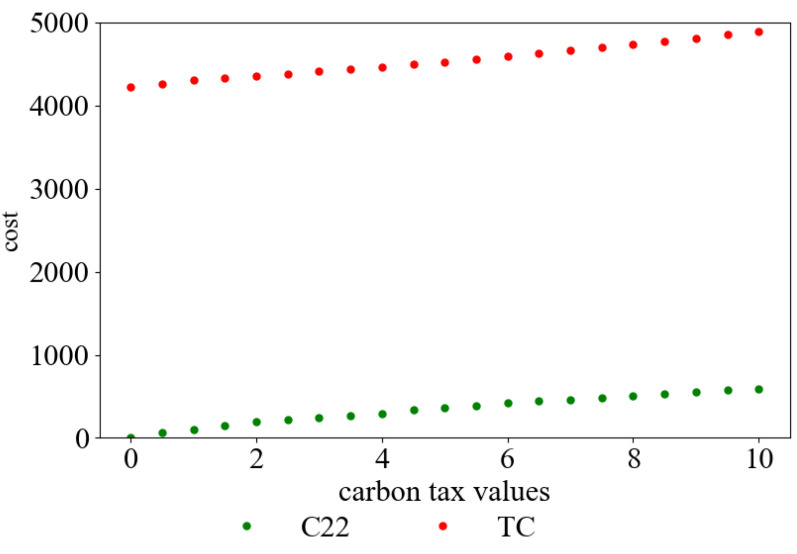
The change in *C*_22_ and TC under different carbon tax values.

**Figure 2:**
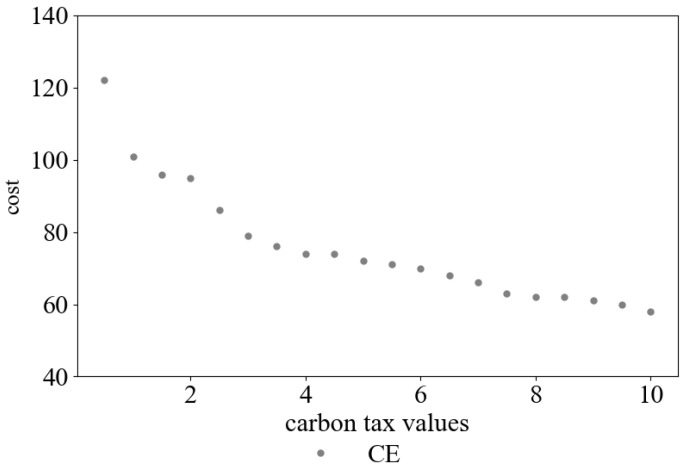
The change in CE under different carbon tax values.

According to the data in Table 2, the curve of carbon emission cost and total cost with different carbon tax values can be obtained. This is shown in Figure 1. Additionally, the curve of carbon emission cost with different carbon tax values is shown in Figure 2.

As can be seen from Table 2, and Figure 1 and Figure 2, with the increase in carbon tax, the carbon emission cost paid by the company rises from CNY 0 to CNY 584, and the total distribution cost rises from CNY 4223 to CNY 4894, persistently. Meanwhile, carbon emission is significantly reduced from 132 kg to 58 kg. This shows that increasing carbon tax can effectively restrict the carbon emission behavior of the distribution companies. It leads them to change their distribution plans and helps to reduce carbon emission in the environment.

It can be further learned from Table 2 that with the continuous increase in carbon tax value, the distribution company’s economic benefits will become worse, and carbon emission will not be significantly reduced. Obviously, if carbon emission is strictly controlled and the value of carbon tax is very large, it is not conducive to the development of the distribution company.

### 4.3. Effect of Freshness-Keeping Cost on CE and TC

In this section, the role of investing in the freshness-keeping cost by the distribution company to control carbon emission, satisfy customers’ satisfaction and improve its economic benefits will be analyzed. The value of carbon tax is set as 0.03 CNY/kg in the subsequent sections of this article.

**Figure 3:**
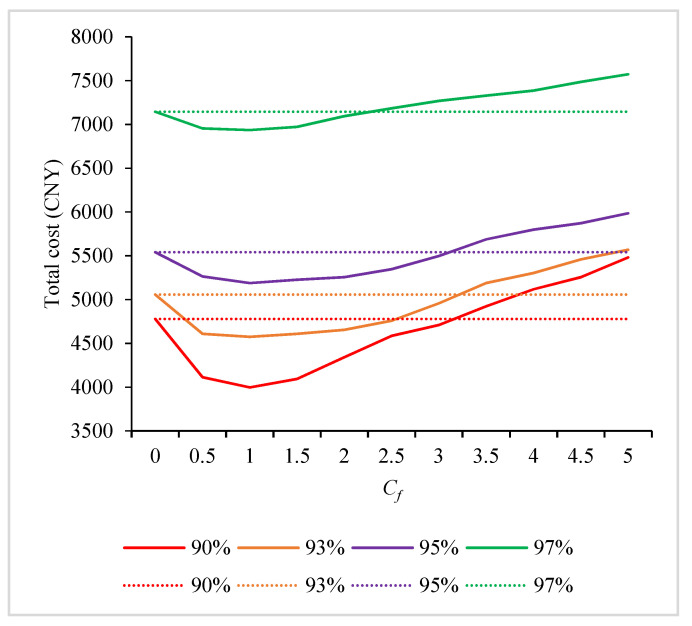
Variation in total cost with *C_f_* under different freshness constraints.

Different freshness constraints and different values of *C_f_* will produce different total costs and carbon emission. The total cost is calculated by setting different values of *C_f_* with different freshness constraints. Under each value, the IACO is run 30 times to obtain the minimum total cost. The results are shown in Figure 3. The solid line in the figure is the total distribution cost calculated according to different values of *C_f_*, and the dotted line is the total distribution cost without investing in the freshness-keeping cost.

It can be seen from Figure 3 that under different freshness requirement constraints, when the investment of *C_f_* is within a certain range, the total cost is less than that of no investment in the freshness-keeping cost. In such cases, investing in the freshness-keeping cost can increase the distribution company’s economic benefits. With the increase in *C_f_*, the increase in the freshness-keeping cost is greater than the decreasing trend of other costs. In such cases, the total cost shows an upward trend, and the economic benefits of the company continue to decrease. When the value of *C_f_* exceeds a certain range, the total distribution cost is larger than that of no investment in the freshness-keeping cost. Thus, investing a large amount in the freshness-keeping cost is not a better choice. For different freshness requirement constraints of customers, the distribution company needs to adjust the amount invested in the freshness-keeping cost.

It can be further seen from Figure 3 that when *C_f_* is less than 1.0, the total cost will be reduced. Therefore, the change in carbon emission before and after investing in the fresh-keeping cost is further analyzed. The relevant results are shown in Table 3.

It can be seen from Table 3 that with the improvement in the freshness-keeping cost, the total distribution cost of the company and carbon emission decrease at the same time. Under the four different freshness constraints, the total cost and carbon emission constantly decrease with the investment in the freshness-keeping cost. As the freshness constraint reflects the demand of customers, the customers’ satisfaction is also improved.

Therefore, the measures to improve the freshness of fresh agricultural products can be beneficial to the company, customers and environment.

### 4.4. Effectiveness Analysis of the IACO

In order to verify the effectiveness of the IACO proposed in this paper, it is compared with the traditional ACO and the A* algorithm. The traditional ACO does not make any modification to the heuristic factor, state-transition strategy or pheromone-update mechanism, and the value of the pheromone volatility coefficient will not change. The movement direction of the A* algorithm is the direction with the smallest total cost. Taking the R108 dataset as the experimental data, the three algorithms are used to solve the model, with freshness constraint values of 90% and 95%. The minimum total cost obtained by using the three algorithms to solve 30 times are shown in Table 4.

It can be seen from Table 4 that the IACO can help the distribution company obtain a smaller total cost (*TC*). When the freshness constraint is 90%, the total cost obtained by the IACO is 8.3% and 21.2% less than that obtained by the ACO and A*, respectively. Meanwhile, it is 2.2% and 17.1% less than that obtained by the ACO and A* when the freshness constraint is 95%. Under the two freshness constraints, the fuel consumption and carbon emission cost (*C*_2_), refrigeration cost and freshness-keeping cost (*C*_3_) obtained by the IACO are smaller than those obtained by the ACO and A*. This indicates that the IACO can help the companies make better distribution plans to reduce fuel consumption and carbon emission costs. This also responds to the call for energy conservation and carbon emission reduction, which is vital to distribution companies.

**Figure 4:**
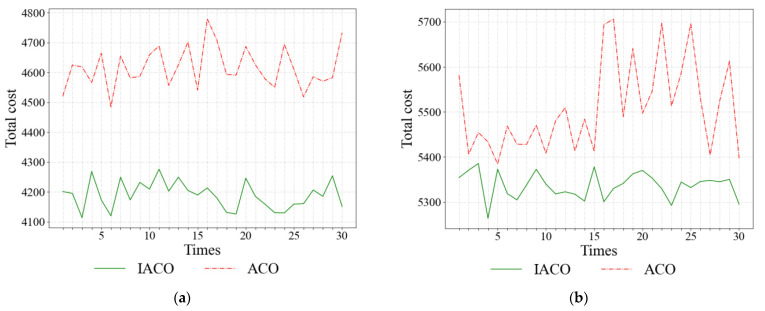
Results of different algorithms under different freshness constraints: (**a**) under 90%, (**b**) under 95%.

The statistical results of all solutions are shown in Table 5. Additionally, the 30 results obtained by IACO and ACO are shown in Figure 4. It also can be seen from the data in Table 5 and Figure 4 that the IACO has a better effect and higher stability than the ACO and A*.

## 5. Conclusions

The most important driving factor of global climate change today is carbon emission and other greenhouse gases emitted by human activities, which is one of the most urgent challenges of current worldwide environmental problems. Aiming to solve the problem of unrestricted carbon emission in the distribution process of cold-chain logistics, we attempt to reduce carbon emission by increasing carbon tax and improving the freshness of fresh agricultural products. A VRP model is established to control carbon emission, improve the freshness of the fresh agricultural products and satisfy customers, based on the relevant research on cold-chain logistics. An improved ant-colony optimization (IACO) is designed to solve the model efficiently. The results of the example calculus show that: (1) Increasing carbon tax values can restrain the carbon emission behavior of the distribution companies. They may avoid paying a high level of carbon emission cost, and achieve the goal of protecting the environment; (2) with the improvement in freshness, the total cost and carbon emission of the distribution companies will be reduced, the customers’ satisfaction will be improved and cargo damage will be reduced; and (3) the IACO designed in this paper has better effects in solving the complex VRP model.

This paper provides a decision-making reference for distribution companies to control carbon emission: (1) The government or management departments can formulate appropriate carbon tax values to reach a balance between reducing carbon emission to protect the environment and promoting the companies’ development; (2) companies can also reduce carbon emission by investing in freshness-keeping costs while improving their own economic benefits and their customers’ satisfaction.

Future research can also investigate open VRP and VRP with various vehicles in depth, and continue to work on exploring other options for reducing carbon emission in the daily operations of distribution companies in cold-chain logistics. Regarding algorithm design, the other heuristic algorithms can also be tried for different situations. In the face of large-scale VRP, some deep learning algorithms such as convolutional neural networks (CNN) can also be considered to obtain better solutions.

## Figures and Tables

**Table 1:** Values of parameters in the model and optimization.

Parameter	Value	Parameter	Value	Parameter	Value
*f_k_*	200 CNY/veh	*c_fuel_*	5.41 CNY/L	*Q_m_*	100
*Q*	2000 kg	*η*_1_	0.01	*α*	1
*P*	12 CNY/kg	*η*_2_	0.02	*β*	3
*ω*	2.669 kg/L	*ε*_1_	10 CNY/h	*q*_0_	0.6
*a*	5 CNY/h	*ε*_2_	10 CNY/h	*NC*_max_	100
*b*	12 CNY/h	*ρ*_0_	0.165 L/km	*M*	35

**Table 2:** Experimental results under different carbon tax values.

*υ* (CNY/kg)	*C*_22_	*TC* (CNY)	*CE* (kilograms)
0.00	0	4223	0
0.25	33	4243	132
0.50	62	4264	122
0.75	88	4286	117
1.00	101	4309	101
1.50	144	4333	96
2.00	191	4358	95
2.50	216	4384	86
3.00	238	4411	79
3.50	266	4439	76
4.00	297	4468	74
4.50	334	4498	74
5.00	362	4529	72
5.50	391	4561	71
6.00	423	4594	70
6.50	448	4628	68
7.00	463	4663	66
7.50	479	4699	63
8.00	501	4736	62
8.50	529	4774	62
9.00	556	4813	61
9.50	576	4853	60
10.00	584	4894	58

Abbreviations: *TC*—total cost (CNY); *CE*—carbon emission (kilograms). The meanings of relevant abbreviations in subsequent parts are the same as in this table.

**Table 3:** TC and CE corresponding to different freshness constraints and different *C_f_* values.

Freshness Constraint	*C_f_*	*TC*	*CE*
90%	0.0	4778	56
	0.5	4113	52
	1.0	3996	51
93%	0.0	5056	69
	0.5	4609	63
	1.0	4575	60
95%	0.0	5541	92
	0.5	5263	85
	1.0	5188	82
97%	0.0	7144	121
	0.5	6954	108
	1.0	6934	104

**Table 4:** Comparison of different algorithms with the minimum total cost.

Freshness Constraint	Algorithm	*TC*	*C*_1_ and *C*_5_	*C*_2_	*C*_3_	*C*_4_
90%	IACO	4113.73	1598.82	1018.03	554.29	942.58
	ACO	4484.45	1788.42	1253.10	584.22	858.71
	A*	5219.41	1753.06	1563.05	673.76	1429.54
95%	IACO	5263.84	3170.29	1196.00	445.51	452.04
	ACO	5384.30	3137.36	1303.40	466.41	477.13
	A*	6350.71	3422.56	1646.07	547.94	734.14

**Table 5:** Comparison of different algorithms with all the results.

Freshness Constraint	Algorithm	Average	Minimum	Maximum	Standard Deviation	Coefficient of Variation
90%	IACO	4189.24	4113.73	4276.35	45.40	0.0108
	ACO	4616.37	4484.15	4780.15	68.29	0.0148
	A*	5219.41	5219.41	5219.41	0	0
95%	IACO	5336.72	5263.84	5385.50	28.66	0.0054
	ACO	5509.94	5384.30	5706.33	98.27	0.0178
	A*	6350.71	6350.71	6350.71	0	0

## Data Availability

The data presented in this study are available on request from the corresponding author.
